# The Need of a Neonatal Preparation for Chagas Disease

**DOI:** 10.1371/journal.pmed.0020387

**Published:** 2005-11-29

**Authors:** Sergio Sosa-Estani, Jose M Belizan, Fernando Althabe, Aldofo Rubinstein

**Affiliations:** **1**Institute for Clinical Effectiveness and Health PolicyBuenos AiresArgentina

We have read about the efforts and initiatives related to the design of drugs for parasitic diseases in McKerrow's article [1] with interest and expectation. One of the pressing needs in this area is for a neonatal preparation for Chagas disease.

Satisfactory achievements have been made in Argentina in relation to the transmission of the disease by vectors and through blood transfusion [2,3]. Vertical transmission is now the great challenge in eradicating Chagas disease. Around 800–1,300 neonates infected with Trypanosmoma cruzi are born every year in our country [4]. Almost 99% of all births occur in hospital, thus allowing the detection of infants born with parasites immediately after birth. The initiation of treatment of these neonates before they and their mothers leave the hospital is a good strategy to obtain high treatment coverage. The later attendance of mothers with their children to health-care facilities is quite unpredictable and irregular. Also, it is difficult to link information about maternal and neonatal parasitic status obtained at birth with later attendance at other health-care facilities.

It would, therefore, be of great value to have a neonatal preparation for the treatment of Chagas disease. There is currently no neonatal or infant preparation available. Instead, one of the two available adult preparations (nifurtimox or benznidazol) is mashed and diluted at local level in order to be administered to newborns and infants. It is easy to understand the difficulties and uncertainties that these procedures involve.

We hope that in the agenda of the several initiatives mentioned in this article [1] the development of a neonatal preparation for Chagas disease could be considered. It would benefit many infants every year.
